# Pain treatment practices and their impact on patient satisfaction in an Ethiopian Emergency Department: a prospective observational study

**DOI:** 10.1016/j.afjem.2026.100964

**Published:** 2026-03-21

**Authors:** Demmelash Gezahegn Nigatu, Mikiyas G. Teferi, Getaw W. Hassen, Tigist Zewdu, Aklilu Azazh

**Affiliations:** a Department of Emergency Medicine and Critical Care, School of Medicine, College of Health Sciences, Addis Ababa University, Addis Ababa, Ethiopia; b School of Medicine, College of Health Sciences, Addis Ababa University, Addis Ababa, Ethiopia

**Keywords:** Pain management, Emergency department, Patient satisfaction, Analgesics, Ethiopia

## Abstract

**Introduction:**

Undertreatment of pain remains a pervasive challenge in emergency departments (EDs) worldwide, reflecting gaps between patient-reported pain and analgesic prescribing and administration practices. This study evaluates pain treatment practices and examines their effect on patient satisfaction at the Tikur Anbessa Specialized Hospital ED in Ethiopia.

**Methods:**

A single-center, prospective observational study assessed pain severity using the Numeric Rating Scale (NRS) at triage and 2–4 h after ED arrival, analgesic prescribing and administration practices, and patient satisfaction using a structured questionnaire among adults presenting with acute or subacute pain.

**Results:**

A total of 106 patients were enrolled, of whom 84 (79.2%) desired analgesia at presentation. Analgesics were prescribed to 54 patients (51.0%) and administered to 44 (41.5%). Tramadol was the most commonly prescribed analgesic (*n* = 34, 63.0%). Overall satisfaction with pain management was reported by 73 patients (68.9%). In multivariable logistic regression, independent predictors of satisfaction were daytime presentation (aOR 8.09, 95% CI 1.48–44.20, *p* = 0.016), repeat NRS pain score (per 1-point increase: aOR 0.69, 95% CI 0.57–0.83, *p* < 0.001), receipt of analgesics in the ED (aOR 4.12, 95% CI 1.68–10.10, *p* = 0.002), and subjective improvement in pain (aOR 5.89, 95% CI 2.45–14.15, *p* < 0.001).

**Conclusion:**

Pain remains frequently undertreated in this tertiary ED, with a substantial gap between patient-reported need and analgesic delivery. Nevertheless, most patients reported satisfaction with pain management, highlighting the limitations of satisfaction as a sole quality indicator. These findings underscore the need for system-level interventions to strengthen pain assessment, timely analgesic administration, and reassessment in resource-constrained emergency care settings.

## Introduction

Pain is an essential biological signal indicating actual or potential tissue damage, prompting protective and remedial actions. It is widely recognized as the most common presenting complaint in emergency departments (EDs) worldwide, constituting approximately 60–80 % of all visits [1,2]. Despite its prevalence, pain remains poorly managed in many settings, particularly in low- and middle-income countries, where healthcare resources and trained personnel are limited [3,4]. Inadequate pain control, also known as oligoanalgesia, contributes to increased patient suffering, psychological distress, and poorer health outcomes, including prolonged hospital stays and progression to chronic pain [5,6]. The World Health Organization (WHO) has emphasized pain management as a fundamental human right, yet access to effective analgesia remains inadequate for billions globally [7].

Effective pain management relies on proper assessment using validated tools such as the Numeric Rating Scale (NRS) and Visual Analogue Scale (VAS). However, the uptake of pain assessment protocols is inconsistent across many African EDs due to factors such as high patient volume, limited training, and lack of clinical guidelines [8,9]. Concerns over opioid misuse, side effects, and regulatory restrictions further limit analgesic availability and physician willingness to prescribe opioids [10]. Studies reveal that even when patients express a clear desire for pain relief, analgesic prescription and administration rates are suboptimal. This gap between need and treatment is compounded by systemic barriers like overcrowding, insufficient drug supply, and inadequate staffing [1].

Patient satisfaction with emergency care is increasingly recognized as a vital outcome measure, with pain relief playing a critical role. Evidence indicates that improved pain control enhances patient satisfaction, adherence to care, and overall perceptions of hospital quality [11]. Nevertheless, data describing the relationship between pain treatment practices and satisfaction in sub-Saharan Africa are limited. Ethiopia, in particular, has a paucity of robust, prospective data exploring these interactions in the emergency care context [12]. Understanding pain management and its impact on patient satisfaction is essential to guide tailored interventions within this resource-constrained environment.

This study prospectively assessed pain treatment practices and their impact on patient satisfaction among adults presenting with pain to the ED at Tikur Anbessa Specialized Hospital (TASH) in Addis Ababa, Ethiopia.

## Methods and materials

### Study design

This was a single-centre, prospective observational study conducted to assess pain management practices and patient satisfaction among adults presenting with pain to the ED.

### Study setting

The study was conducted in the ED of TASH, Ethiopia’s largest tertiary referral and teaching hospital in Addis Ababa, which receives referrals from across the country. The ED operates 24 h a day and manages approximately 18,000–20,000 patient visits annually, primarily serving patients referred from peripheral facilities rather than self-presenting. Patients are triaged using the Triage Early Warning System (TEWS) into color-coded urgency categories. The ED also serves as a holding area for patients awaiting inpatient beds or specialist consultation.

### Study population and sampling

The study population comprised adult patients aged 18 years or older who presented to the ED with pain of recent onset, defined as pain duration of three months or less. This timeframe was selected to capture acute and subacute pain presentations typically managed in emergency care settings and to exclude chronic pain syndromes requiring specialised long-term management.

Patients were eligible if they had NRS of 1–10 at triage and were able to participate in follow-up assessment. Exclusion criteria included refusal to provide consent, altered mental status or neuropsychiatric impairment precluding reliable assessment, immediate life-threatening conditions requiring urgent resuscitation, discharge before follow-up assessment, or death on arrival. Consecutive eligible patients presenting during the study period were enrolled. No a priori sample size calculation was performed.

### Data collection

Data were collected continuously over a five-day period, covering all hours of the day. Data collectors included one trained nurse and one medical intern, who received prior instruction on study procedures, consent processes, and use of study tools.

A structured 28-item questionnaire was administered twice for each participant: at initial triage and again 2–4 h after ED arrival. The follow-up interval was selected to allow sufficient time for clinical evaluation and initiation of treatment while remaining feasible within routine ED workflow. The timing of reassessment was standardised within this window but could vary slightly depending on patient flow and clinical circumstances. The questionnaire captured sociodemographic information, pain characteristics (including location, duration, and severity using the NRS), pre-ED analgesic use within the preceding 12 h, desire for analgesics at presentation, analgesic prescription and administration during ED stay, and patient-reported satisfaction with pain management measured using a 5-point Likert scale. Satisfaction responses were dichotomised for analysis. Prior to data collection, the questionnaire was piloted on a small number of patients to assess clarity and feasibility. Minor wording adjustments were made based on feedback to improve comprehension; no substantive changes to content were required.

Pain severity was categorised as mild (NRS 1–3), moderate (NRS 4–7), or severe (NRS >7). While NRS assessment was conducted as part of the study protocol rather than routine clinical practice, data collectors informed the treating team if severe pain (NRS >7) was identified, in accordance with ethical considerations. The full structured 28-item questionnaire used for data collection is provided as Supplementary Material 1.

### Data analysis

Data were entered into Microsoft Excel and analysed using SPSS version 21. Descriptive statistics were used to summarise participant characteristics, pain severity, analgesic use, and satisfaction outcomes. Normality of continuous variables was assessed using visual inspection of histograms and the Shapiro–Wilk test. Most continuous variables were non-normally distributed and are therefore reported as medians with interquartile ranges (IQRs), including NRS. Normally distributed continuous variables, where applicable, were summarised using means with standard deviations. Categorical variables are reported as frequencies and percentages. Patient satisfaction with pain management was assessed using a 5-point Likert scale with the following response options: 1 = very dissatisfied, 2 = dissatisfied, 3 = neutral, 4 = satisfied, and 5 = very satisfied. For analytical purposes, satisfaction responses were dichotomised into satisfied (Likert scores of 4–5) and not satisfied (Likert scores of 1–3), consistent with prior ED satisfaction studies [1,4]. This dichotomised variable was used in all subsequent regression and comparative analyses, while the original 5-point Likert categories were retained for descriptive reporting and for non-parametric comparisons of scores across satisfaction levels presented in the supplementary material.

Univariable comparisons between groups were performed using chi-square tests for categorical variables and the Mann–Whitney U test for non-normally distributed continuous variables. Comparisons of NRS across three or more independent groups were conducted using the Kruskal–Wallis test. Logistic regression modelling was used to examine factors independently associated with physician analgesic prescription and overall patient satisfaction, with results presented as odds ratios (ORs) and 95 % confidence intervals (CIs).

For multivariable logistic regression, variable selection followed a two-step approach. First, variables with *p* < 0.20 in univariable analyses were considered candidates for inclusion. Second, final model selection was guided by clinical relevance, statistical significance (*p* < 0.05), and minimization of the Akaike Information Criterion (AIC). Adjusted odds ratios (aORs) with 95 % confidence intervals are reported for the final multivariable models, and the variables retained in each model are specified in the corresponding table footnotes. Given the number of variables examined, the potential for Type I error due to multiple comparisons should be considered. Formal adjustment methods such as Bonferroni correction were not applied, as several key associations demonstrated strong statistical significance. Associations with marginal p-values should therefore be interpreted cautiously as exploratory and hypothesis-generating rather than confirmatory.

### Ethical considerations

Ethical approval was obtained from the Departmental Research Committee of Emergency Medicine, Addis Ababa University College of Health Sciences (Ref: AAU/ECCM-016/2019). Verbal informed consent was obtained from all participants after explanation of the study purpose, procedures, risks, and voluntary nature of participation. Participant confidentiality was maintained by anonymising data and restricting access to study records.

## Results

**Fig. 1:**
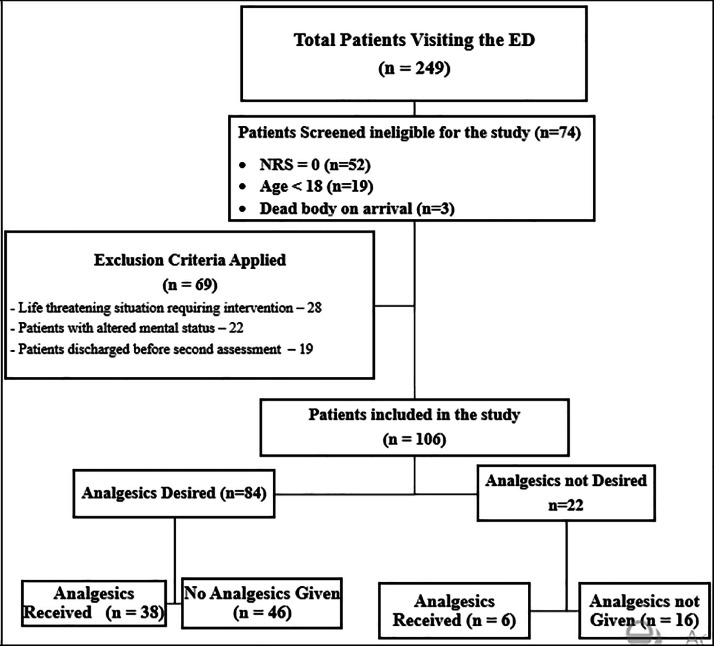
CONSORT diagram of Patient allocation through the study.

A total of 175 patients were eligible for this study, of whom 69 patients were excluded based on the exclusion criteria, with 106 patients ultimately enrolled (Fig. 1).

### Participant characteristics

Male patients accounted for 57.5 % of the sample (*n* = 61), with a male-to-female ratio of 1.3:1. The mean age was 42.5 years (SD=16.3). Most patients presented during daytime working hours, accounting for 69.8 % (*n* = 74) of presentations. The majority of patients, 67.9 % (*n* = 72), paid out-of-pocket for medications. There were 61 (57.5 %) patients referred by primary healthcare providers, while 45 (42.5 %) were self-referrals. Most patients were triaged to the Yellow/Green category, accounting for 84.0 % (*n* = 89), followed by the Orange category at 13.2 % (*n* = 14) and the Red category at 2.8 % (*n* = 3). Chronic illness was present in 64.2 % (*n* = 68) of participants, with malignancy being the most common underlying condition (*n* = 36), followed by other chronic medical conditions. The primary presenting complaint categories were medical emergencies in 35.8 % (*n* = 38), oncologic emergencies in 31.1 % (*n* = 33), trauma in 19.8 % (*n* = 21), and surgical emergencies in 13.2 % (*n* = 14) of patients (Table 1).

**Table 1:** Demographic and clinical characteristics of patients by desire for analgesia (*n* = 106).

Characteristic	Desire for Analgesia – Yes (*n* = 84), n ( %) or Median (IQR)	Desire for Analgesia – No (*n* = 22), n ( %) or Median (IQR)	Univariable p-value*
**Sex**			0.43
**Male**	50 (59.5)	11 (50.0)	
**Female**	34 (40.5)	11 (50.0)	
**Age, years**	42 (31–53)	44 (31–58)	0.64
**Duration of pain, days**	2 (1–6)	4 (2–10)	0.037
**Time of presentation**			0.74
**Daytime**	58 (69.0)	16 (72.7)	
**Night-time**	26 (31.0)	6 (27.3)	
**Triage category (TEWS)**			0.67
**Red**	3 (3.6)	0 (0.0)	
**Orange**	11 (13.1)	3 (13.6)	
**Yellow/Green**	70 (83.3)	19 (86.4)	
**Mode of referral**			0.43
**Self-referral**	34 (40.5)	11 (50.0)	
**Referred by primary care**	50 (59.5)	11 (50.0)	
**Payment method**			0.12
**Free care**	30 (35.7)	4 (18.2)	
**Out-of-pocket**	54 (64.3)	18 (81.8)	
**Pain location**			0.75
**Head and neck**	9 (10.7)	2 (9.1)	
**Chest**	23 (27.4)	3 (13.6)	
**Abdomen**	17 (20.2)	8 (36.4)	
**Pelvis**	8 (9.5)	2 (9.1)	
**Upper extremity**	10 (11.9)	3 (13.6)	
**Lower extremity**	12 (14.3)	3 (13.6)	
**Flank**	3 (3.6)	0 (0.0)	
**Back**	2 (2.4)	1 (4.5)	
**NRS at triage**	7 (5–8)	4 (2–6)	<0.001
**Pre-ED analgesic use**			0.09
**No**	64 (76.2)	17 (77.3)	
**Yes**	20 (23.8)	5 (22.7)	
**Category of illness**			0.71
**Medical**	29 (34.5)	9 (40.9)	
**Surgical**	10 (11.9)	4 (18.2)	
**Trauma**	18 (21.4)	3 (13.6)	
**Oncologic**	27 (32.1)	6 (27.3)	
**Underlying chronic illness**			0.72
**No**	30 (35.7)	8 (36.4)	
**Yes**	54 (64.3)	14 (63.6)	

⁎ Univariable p-values derived from chi-square tests for categorical variables (overall association across categories) and Mann–Whitney U tests for continuous variables. TEWS - Triage Early Warning Score; NRS -numeric rating scale for pain; ED emergency department

### Pain characteristics

The median duration of pain prior to ED presentation was 3 days (IQR 1–7). At initial triage assessment, the overall median NRS was 6 (IQR 4–7) on the NRS. Pain severity differed by desire for analgesia, with higher scores among patients requesting analgesics (median NRS 7 [IQR 5–8] vs. 4 [IQR 2–6], *p* < 0.001) (Table 1). Moderate pain (NRS 4–7) was present in 70 patients (66.0 %), severe pain (NRS >7) in 20 (18.9 %), and mild pain (NRS 1–3) in 16 (15.1 %). Although 57.5 % (*n* = 61) of patients were referred by primary care providers, 77.4 % (*n* = 82) had not received analgesics prior to ED presentation. There was no association between patient mode of referral and pre-ED analgesics intake (*p* = 0.134).

### Pre-ED analgesic use and desire for pain relief

Among the 106 enrolled patients, 84 (79.2 %) expressed a desire for analgesics at initial triage. Patients desiring analgesics had a shorter median duration of pain prior to presentation (2 days [IQR 1–6] vs. 4 days [IQR 2–10], *p* = 0.037). No significant differences were observed between the two groups with respect to age, sex, triage category, time of presentation, mode of referral, payment method, pain location, pre-ED analgesic use, category of illness, or underlying chronic illness (Table 1).

### Analgesic prescription and administration

At initial triage, 84 patients (79.2 %) expressed a desire for analgesia, while 22 (20.8 %) did not. Analgesics were prescribed for 54 patients (51.0 %), whereas 52 patients (49.0 %) did not receive a prescription. Actual administration of analgesics occurred in 44 patients (41.5 %), while 62 patients (58.5 %) did not receive any analgesic during their ED stay.

When examined by initial preference, among the 84 patients who desired analgesia, 38 (45.2 %) received analgesics and 46 (54.8 %) did not. Among the 22 patients who initially did not desire analgesia, 6 (27.3 %) later received analgesics and 16 (72.7 %) did not. This yielded a total of 44 patients who received analgesics and 62 who did not (Fig. 1).

Among the 62 patients who did not receive analgesics, the primary reason was absence of prescription in 52 cases (83.9 %). The remaining 10 patients (16.1 %) had analgesics prescribed but did not receive them due to non-administration or medication unavailability. Among patients who were prescribed analgesics (*n* = 54), tramadol was the most frequently prescribed agent (*n* = 34, 63.0 %), followed by morphine and paracetamol.

Patients who received analgesics had higher initial scores compared with those who did not (median NRS 7.0 [IQR 5.0–8.0] vs. 5.0 [4.0–7.0], *p* = 0.001). At reassessment 2–4 h after arrival, patients who received analgesics demonstrated greater reduction in pain severity (median NRS 3.0 [2.0–4.0]) compared with those who did not receive analgesics (median NRS 4.0 [3.0–6.0], *p* = 0.008) (Table 2).

**Table 2:** Initial and repeat numerical rating scale (NRS) for pain by analgesic receipt (median [IQR], Mann-Whitney U).

NRS	Did Patient take Analgesics?	n	Median (IQR)	Mann‑Whitney U	p‑value
**Initial NRS**	Yes	44	7.0 (5.0–8.0)	833.5	0.001
	No	62	5.0 (4.0–7.0)		
**Repeat NRS**	Yes	44	3.0 (2.0–4.0)	958.5	0.008
	No	62	4.0 (3.0–6.0)		

### Factors associated with physician analgesic prescription

In univariable analyses, physician analgesic prescription was significantly associated with patient-initiated requests for analgesics (*p* < 0.001), daytime presentation (*p* = 0.025), higher initial NRS at triage (median 6 [IQR 5–8] vs. 5 [IQR 4–7], *p* = 0.005), and category of illness (*p* = 0.004) (Table 3). No significant associations were observed between analgesic prescription and patient age, sex, duration of pain, triage category, referral source, payment method, pain location, pre-ED analgesic use, or presence of underlying chronic illness. Desire for analgesia’ reflects patient preference expressed during structured triage assessment, whereas ‘patient requested analgesics’ refers to whether the patient explicitly asked a clinician for pain medication during the ED encounter. These represent distinct constructs and were analysed separately.

**Table 3:** Univariable associations with physician analgesic prescription in the Emergency Department (*n* = 106).

Predictor	Analgesics Prescribed, n ( %) or Median (IQR)	Not Prescribed, n ( %) or Median (IQR)	Univariable p-value*
Sex			0.72
Male (*n* = 61)	32 (52.5)	29 (47.5)	
Female (*n* = 45)	22 (48.9)	23 (51.1)	
Age, years	45 (32–56)	38 (27–51)	0.15
Duration of pain, days	3 (1–7)	3 (1–7)	0.91
Time of presentation			0.025
Daytime (*n* = 74)	43 (58.1)	31 (41.9)	
Night-time (*n* = 32)	11 (34.4)	21 (65.6)	
Triage category (TEWS)			0.82
Red (*n* = 3)	1 (33.3)	2 (66.7)	
Orange (*n* = 14)	7 (50.0)	7 (50.0)	
Yellow/Green (*n* = 89)	46 (51.7)	43 (48.3)	
Mode of referral			0.28
Self-referral (*n* = 45)	19 (42.2)	26 (57.8)	
Referred from primary care (*n* = 61)	35 (57.4)	26 (42.6)	
Payment method			0.33
Free care (*n* = 34)	15 (44.1)	19 (55.9)	
Out-of-pocket (*n* = 72)	39 (54.2)	33 (45.8)	
Pain location			0.21
Head and neck (*n* = 11)	2 (18.2)	9 (81.8)	
Chest (*n* = 26)	12 (46.2)	14 (53.8)	
Abdomen (*n* = 26)	12 (46.2)	13 (53.8)	
Pelvis (*n* = 10)	5 (50.0)	5 (50.0)	
Upper extremity (*n* = 13)	10 (76.9)	3 (23.1)	
Lower extremity (*n* = 15)	9 (60.0)	6 (40.0)	
Flank (*n* = 3)	2 (66.7)	1 (33.3)	
Back (*n* = 3)	2 (66.7)	1 (33.3)	
NRS at triage	6 (5–8)	5 (4–7)	0.005
Pre-ED analgesic use			0.56
Yes (*n* = 24)	14 (58.3)	10 (41.7)	
No (*n* = 82)	40 (48.8)	42 (51.2)	
Patient requested analgesics			<0.001
Yes (*n* = 52)	47 (90.4)	5 (9.6)	
No (*n* = 54)	7 (13.0)	47 (87.0)	
Category of illness			0.004
Medical (*n* = 38)	11 (28.9)	27 (71.1)	
Surgical (*n* = 14)	8 (57.1)	6 (42.9)	
Trauma (*n* = 21)	16 (76.2)	5 (23.8)	
Oncologic (*n* = 33)	19 (57.6)	14 (42.4)	
Underlying chronic illness	31 (45.6)	37 (54.4)	0.14

⁎ Univariable p-values derived from chi-square tests for categorical variables (overall association across categories) and Mann–Whitney U tests for continuous variables.

### Patient satisfaction with pain management

At reassessment 2–4 h after presentation, 62 patients (58.5 %) reported subjective improvement in their condition, 38 (35.8 %) reported no change, and 6 (5.7 %) reported worsening symptoms. The overall median repeat NRS was 4.0 (IQR 2.0–5.0).

Overall satisfaction with pain management was reported by 73 patients (68.9 %), while 33 patients (31.1 %) were not satisfied. Distribution of NRS across satisfaction categories and non-parametric comparisons are presented in **Supplementary Table S1.**

In the multivariable logistic regression model using dichotomised patient satisfaction (satisfied vs not satisfied), independent predictors of patient satisfaction were daytime presentation (aOR=8.09, 95 % CI: 1.48–44.2, *p* = 0.016), repeat NRS (per 1-point increase: aOR=0.69, 95 % CI: 0.57–0.83, *p* < 0.001), patient receiving analgesics in the ED (aOR=4.12, 95 % CI: 1.68–10.10, *p* = 0.002), and subjective improvement in pain (aOR=5.89, 95 % CI: 2.45–14.15, *p* < 0.001) (Table 4). Triage score, initial NRS, pain duration, and patient age showed no association with overall satisfaction (**Supplementary Table S2**).

**Table 4:** Multivariable logistic regression model of predictors of overall satisfaction with pain management (*n* = 106).

Predictor	Level / Unit	aOR (95 % CI)	p-value
**Time of presentation**	Daytime vs. Night-time	8.09(1.48–44.20)	0.016
**Repeat NRS**	Per 1-point increase	0.69 (0.57–0.83)	<0.001
**Patient received analgesics in ED**	Yes vs. No	4.12(1.68–10.10)	0.002
**Subjective change in pain**	Improved vs. Same/Worse	5.89(2.45–14.15)	<0.001

### ED length of stay

Mean ED length of stay was 51.36 h (SD=2.36), with a range of 4 h to 13 days. Disposition within 24 h occurred for 52 (49.1 %) patients. Fifty-three (50.0 %) patients were discharged home, 42 (39.6 %) were admitted for inpatient care, and 6 (5.7 %) were transferred to other hospitals. The remaining five patients (4.7 %) left against medical advice or had undocumented disposition at the time of data collection. Referral source was independently associated with ED length of stay <24 h (OR=3.3, 95 % CI: 1.42–7.66, *p* = 0.005). Higher TEWS score (*p* = 0.032) and higher initial NRS (*p* = 0.036) were associated with longer ED stays. Patient age, pain duration, repeat NRS, and overall satisfaction showed no association with length of stay.

## Discussion

This study provides prospective evidence that pain management remains suboptimal among adults presenting to the ED at TASH, with a clear gap between patient-reported pain and analgesic prescribing and administration. Although many patients experienced improvement in pain intensity during ED care and reported satisfaction, a substantial proportion did not receive analgesics despite expressing a desire for pain relief. These findings highlight important opportunities to strengthen pain assessment and treatment processes in resource-constrained ED settings [3].

The patient population in this study represents an atypical ED case-mix, reflecting the tertiary referral role and specialized functions of TASH. A substantial proportion of patients presented with oncologic emergencies (31.1 %) and chronic illness–related pain, and the mean duration of pain prior to ED presentation was nearly five days. This differs from typical ED populations in high-income settings, which are often dominated by acute trauma, ischemic, or short-duration pain presentations [2,4]. The predominance of referred patients, high burden of chronic disease, and delayed presentation likely influenced both pain severity and expectations of care, and may partially explain the observed gap between patient-reported pain and analgesic delivery.

The observed gap between patient-reported pain and analgesic delivery is consistent with prior studies from sub-Saharan Africa and other low- and middle-income settings, which have documented persistent oligoanalgesia in emergency care [13,14]. Similar to reports from Ethiopia and neighboring countries, analgesic prescribing was inconsistent and often delayed, particularly for patients with moderate to severe pain [[14], [15], [16], [17]]. While our findings align with this broader literature, this study adds value by prospectively assessing pain severity, patient preferences, and satisfaction within the same ED encounter, allowing a more integrated view of pain care delivery at TASH [18,19].

Importantly, this study does not identify clinician knowledge, attitudes, or hesitancy as causal explanations for undertreatment, as these factors were not directly measured. Instead, the findings describe observable patterns in pain assessment, prescribing, and administration within routine ED workflows [20,21]. The lack of consistent alignment between patient-reported pain severity and analgesic use suggests that systematic approaches to pain assessment and reassessment may be insufficiently integrated into routine practice [3,20]. This interpretation supports system-level rather than individual-level explanations and aligns with evidence from other EDs showing that protocol-driven pain management improves consistency of care [[21], [22], [23]].

Analgesic prescription was associated with patient-initiated requests and daytime presentation, although the estimate for patient requests was imprecise and should be interpreted cautiously [24,25]. These associations suggest that contextual and process-related factors may influence pain management decisions in the ED. Similar patterns have been reported elsewhere, where workload, staffing patterns, and clinical flow affect the timing and likelihood of analgesic administration. However, given the observational design and limited study period, these associations should be considered exploratory rather than definitive [4,5,13,26].

Despite the high prevalence of undertreatment, overall patient satisfaction with pain management was moderate [13]. This apparent disconnect has been described in other low-resource settings and may reflect adjusted patient expectations, perceived attentiveness of care, or improvement in symptoms over time rather than optimal analgesic delivery. These findings reinforce the importance of using objective quality indicators, such as timely pain assessment and reassessment, rather than patient satisfaction alone to evaluate pain management performance in EDs [6,8,[22], [23], [24], [25], [26]].

Taken together, the findings underscore the need for structured, system-level interventions to improve pain care at TASH and similar EDs. Standardized pain assessment, timely initiation of analgesia, and routine reassessment are widely recommended and have demonstrated effectiveness in comparable settings. By focusing on processes of care rather than unmeasured clinician attributes, these interventions offer pragmatic and scalable pathways to improve pain management quality in resource-limited emergency care environments.

### Limitations

This study has several limitations that should be considered when interpreting the findings. Its single-centre design and short data collection period may limit generalisability to other EDs or time periods. The observational design precludes causal inference, and unmeasured confounders may have influenced observed associations. Pain severity and satisfaction were based on patient self-report, which introduces subjectivity and may be influenced by contextual or cultural factors. In addition, pain assessment using the NRS was conducted as part of the study protocol rather than routine clinical practice, which may have affected alignment between measured scores and prescribing decisions. Finally, the analysis involved multiple comparisons, and associations with marginal statistical significance should be interpreted cautiously as exploratory.

## Conclusion

This study demonstrates that pain remains frequently undertreated among adults presenting to a tertiary ED in Ethiopia, despite most patients expressing a desire for analgesia and reporting overall satisfaction with care. The coexistence of persistent oligoanalgesia with acceptable satisfaction highlights a critical quality gap that is unlikely to be addressed through patient feedback alone. These findings underscore the need for system-level approaches to strengthen pain assessment, prescribing, and reassessment practices within resource-constrained emergency care settings.

Immediate improvements should prioritise five evidence-based interventions: structured clinician education on the WHO Analgesic Ladder with emphasis on safe and appropriate opioid use for acute pain; implementation of nurse-led triage-initiated analgesia protocols guided by NRS scores; integration of pain assessment as a routine “fifth vital sign” with scheduled reassessment; ensuring consistent availability of essential analgesics across all levels of the WHO pain ladder; and establishment of regular quality-improvement audits focused on time to analgesia, appropriateness of analgesic selection, and documentation of reassessment. Future research should evaluate these strategies through pragmatic designs, including randomised trials of nurse-initiated analgesia, pre-post studies assessing targeted educational interventions, and multicentre prospective cohorts to characterise variability across Ethiopian EDs and develop context-appropriate quality indicators. Sustained improvement in pain management will require coordinated clinical leadership, protocol-driven care, and continuous monitoring rather than reliance on patient dissatisfaction as a signal for change.

## Dissemination of results

Results were presented to the clinical staff and administration of TASH ED to inform quality improvement initiatives in pain management protocols. Findings will be presented at regional and international emergency medicine conferences to facilitate knowledge exchange and support evidence-based pain management practices across similar resource-limited emergency care settings

## CRediT authorship contribution statement

**Demmelash Gezahegn Nigatu**: Conceptualization, Methodology, Investigation, Data curation, Formal analysis, Writing – original draft, Project administration. **Mikiyas G. Teferi**: Writing – review and editing. **Getaw W. Hassen**: Writing – review and editing, Validation. **Tigist Zewdu**: Conceptualization, Methodology, Supervision, Writing – review and editing. **Aklilu Azazh**: Conceptualization, Methodology, Supervision, Writing – review and editing. All authors approved the final version to be published and agreed to be accountable for all aspects of the work.

## Availability of data and material

The datasets generated and/or analyzed during the current study are available from the corresponding author upon reasonable request.

## Funding

None received

## Declaration of competing interest

The authors declare no conflict of interest.

## Appendix Supplementary materials

Image, application 1
