# Supplementary material for: Pain treatment practices and their impact on patient satisfaction in an Ethiopian Emergency Department: a prospective observational study
Source: Afr J Emerg Med. 2026 Mar 21;16(2):100964. doi: 10.1016/j.afjem.2026.100964 (PMC13221742; doi:10.1016/j.afjem.2026.100964)
Supplement: Supplementary file 1 [file mmc1.docx]

**Questionnaire**

**To be Filled at Triage**

| **PART I** | |  |
| --- | --- | --- |
| 1. | Code ……………. | a. Day time b. Night time |
| 2. | MRN…………….. | Data of triage ……../07/2019 |
|  |  | Time …………………. |

1. **Age (In years) ……………..**

| 4. | Sex | a. Male |  | b. Female |
| --- | --- | --- | --- | --- |
| 5. | Mode of payment | | a. Free | b. Payer |

1. **Mode of referral**
   1. Self, from home
   2. Central Triage
   3. From OPD / Follow up Clinic (Specify)……………………………………….
   4. From public hospital
   5. From private hospital

**PART II**

1. **Duration of the pain?**................................................................
2. **Where do you feel the pain?**

| a. | Head and neck | |  |  |  | e. Upper extremities | | |  |
| --- | --- | --- | --- | --- | --- | --- | --- | --- | --- |
| b. | Chest |  |  |  |  | f. lower extremities | | |  |
| c. | Abdomen | |  |  |  | g. flank | |  |  |
| d. | Pelvis |  |  |  |  | h. back |  |  |  |
| **9. Numeric Pain Score Now?** | | | |  |  |  |  |  |  |
| (1 | 2 | 3 | 4 | 5 | 6 | 7 | 8 | 9 | 10) |
| 10. **Took anti-pain prior to presentation to ER (with-in 12 hours)** | | | | | | | | | a. yes b. no |

1. If yes, what kind of medication?
   1. Paracetamol
   2. NSAIDs (Specify)………………………….
   3. Weak opioids (Specify)…………………………..
   4. Strong opioids (Specify)…………………………..
   5. Others (like nitrates, amitriptyline, triptans) ………………..

12. **Would you like to have anti pain (የህመም ማስታገሻ) now?** a. Yes b. No

1. **If no, reason for refusal of analgesics**
   1. Bearable pain or minimal to no pain
   2. Took analgesics before coming to ER
   3. Needs to settle diagnosis rather than getting symptomatic treatment
   4. Others (specify) ………………………………………….
2. Triage Score (TEWS) ………………..

a. Red b. Orange c. Yellow/green

1. Known chronic illness (if any)
   1. …………………………..b……………………..
2. Category of illness for current presentation
   1. Medical emergency

35

1. Surgical emergency
2. Trauma
3. Oncologic emergency + Hematologic malignancy (Any patient with malignancy coming to ER with its complications)

**To be filled After 2-4 hours of ED Stay**

**PART III**

1. Working diagnosis for current presentation (Clinical impression + After Work up)
   1. …………………………………...
   2. ……………………………………
   3. ……………………………………
   4. ……………………………………

| 2. | Was analgesic prescribed by treating physician? | a. Yes | b. No |
| --- | --- | --- | --- |
| 3. | **Did the patient ask for analgesics to the treating team?** | a. Yes | b. No |
| 4. | Was analgesics given/taken to the patient/by the patient in ER? a. Yes | | b. No |

1. What analgesics, Route and dose?
   1. Paracetamol……………………………
   2. NSAIDs…………………………………
   3. Weak opioids ……………………………
   4. Strong opioids……………………………
   5. Others……………………………………
2. If no to question 4, why?
   1. Analgesics not ordered by treating team
   2. Patient couldn’t afford to buy from hospital pharmacy
   3. Medication not available in hospital pharmacy
   4. Analgesic not yet administered by the treating team
3. **How is your pain (የህመም ስሜት፣ ስቃይ፣ ቃንዛ) now?**
   1. Improved
   2. The same
   3. Worse
4. **Repeat Numeric Rating Score**

0 1 2 3 4 5 6 7 8 9 10

1. **How is your overall pain management in the ER? (በህመም ማስታገሻ ህክምናው ምን ያህል**

**ረክተዋል?)**

1 2 3 4 5

**PART IV**

1. Length of stay in the ER ……………………………………………
2. Disposition/ Outcome of Treatment in ER
   1. Discharged home improved
   2. Went home against medical advice
   3. Admitted to ward
   4. Transferred to other hospital
   5. Died

**Supplementary Table S1.** Distribution and Non-parametric Comparison of Numeric Rating Scale (NRS) Pain Scores Across Patient Satisfaction Categories (5-point Likert scale) (n = 106)

| **Patient Satisfaction Category** | **n (%)** | **Initial NRS at Triage Median (IQR)** | **Repeat NRS at 2–4 Hours Median (IQR)** |
| --- | --- | --- | --- |
| Very dissatisfied | 14 (13.2) | 6.5 (5.0–8.0) | 6.0 (5.0–7.0) |
| Dissatisfied | 19 (17.9) | 6.0 (4.0–7.0) | 5.0 (4.0–6.0) |
| Neutral | 0 (0.0) | – | – |
| Satisfied | 63 (59.4) | 6.0 (4.0–7.0) | 3.0 (2.0–4.0) |
| Very satisfied | 10 (9.4) | 5.5 (4.0–7.0) | 2.0 (1.0–3.0) |
| **Overall comparison (Kruskal–Wallis test)** |  | **H = 6.83, df = 3, p = 0.145** | **H = 42.51, df = 3, p < 0.001** |
| **Pairwise comparisons (Repeat NRS only)** |  |  | Very dissatisfied vs Satisfied: p < 0.001 |
|  |  |  | Very dissatisfied vs Very satisfied: p < 0.001 |
|  |  |  | Dissatisfied vs Satisfied: p < 0.001 |
|  |  |  | Dissatisfied vs Very satisfied: p < 0.001 |
|  |  |  | Satisfied vs Very satisfied: p = 0.072 |

Pain scores at triage and at repeat assessment (2–4 hours) are presented as median (interquartile range) across the five patient satisfaction categories measured using the original 5-point Likert scale. Differences in pain score distributions across satisfaction categories were assessed using the Kruskal–Wallis test. Post-hoc pairwise comparisons were conducted for repeat NRS scores using Dunn’s test with Bonferroni adjustment; only significant comparisons are shown. The original Likert categories were used for these non-parametric comparisons, while dichotomised patient satisfaction (satisfied vs not satisfied) was used for regression analyses in the main manuscript. NRS = Numeric Rating Scale (0–10). df = 3 because the "Neutral" category had no observations (n=0).

**Supplementary Table S2.** Univariable Associations with Dichotomised Patient Satisfaction (Satisfied vs Not Satisfied) (n = 106)

| **Predictor** | **Level / Summary** | **Not satisfied (n=33) n (%) or Median (IQR)** | **Satisfied (n=73) n (%) or Median (IQR)** | **Crude OR (95% CI)** | **p-value** |
| --- | --- | --- | --- | --- | --- |
| **Time of presentation** | Daytime | 10 (30.3) | 64 (87.7) | 8.09 (1.48–44.20) | 0.025 |
|  | Night-time | 23 (69.7) | 9 (12.3) | 1.0 (Ref) |  |
| **Sex** | Male | 18 (54.5) | 43 (58.9) | 1.20 (0.53–2.70) | 0.667 |
|  | Female | 15 (45.5) | 30 (41.1) | 1.0 (Ref) |  |
| **Mode of referral** | Self-referral | 16 (48.5) | 29 (39.7) | 1.0 (Ref) | 0.397 |
|  | Referred | 17 (51.5) | 44 (60.3) | 1.43 (0.63–3.24) |  |
| **Repeat NRS pain score** | Median (IQR) | 5.0 (4.0–6.0) | 3.0 (2.0–4.0) | 0.45 (0.34–0.60)† | <0.001 |
| **Received analgesics in ED** | Yes | 8 (24.2) | 36 (49.3) | 3.00 (1.21–7.46) | 0.018 |
|  | No | 25 (75.8) | 37 (50.7) | 1.0 (Ref) |  |
| **Patient requested analgesics** | Yes | 10 (30.3) | 42 (57.5) | 3.11 (1.31–7.40) | 0.010 |
|  | No | 23 (69.7) | 31 (42.5) | 1.0 (Ref) |  |
| **Subjective change in pain** | Improved | 10 (30.3) | 52 (71.2) | 5.72 (2.38–13.76) | <0.001 |
|  | Same/Worse | 23 (69.7) | 21 (28.8) | 1.0 (Ref) |  |
| **Chronic illness** | Present | 23 (69.7) | 45 (61.6) | 0.70 (0.29–1.69) | 0.432 |
|  | Absent | 10 (30.3) | 28 (38.4) | 1.0 (Ref) |  |
| **TEWS (triage score)** | Median (IQR) | 3 (2–4) | 3 (2–4) | — | 0.282‡ |
| **Age (years)** | Median (IQR) | 43 (30–56) | 42 (31–53) | — | 0.588‡ |

Univariable associations between patient characteristics and dichotomised patient satisfaction with pain management (satisfied vs not satisfied). Categorical variables are presented as n (%) with crude odds ratios (ORs) from univariable logistic regression. Continuous variables are presented as median (IQR) and compared using Mann–Whitney U tests.

**Footnotes**

† Crude OR for repeat NRS represents the odds of satisfaction per 1-point increase in repeat pain score. ‡ p-values for continuous variables derived from Mann–Whitney U tests. Patient satisfaction was dichotomised as satisfied (Likert 4–5) vs not satisfied (Likert 1–3). ED = emergency department; NRS = Numeric Rating Scale (0–10); TEWS = Triage Early Warning System.
